# Alternative splicing acts as an independent prognosticator in ovarian carcinoma

**DOI:** 10.1038/s41598-021-89778-0

**Published:** 2021-05-17

**Authors:** Yan Ouyang, Kaide Xia, Xue Yang, Shichao Zhang, Li Wang, Shan Ren, Houming Zhou, Yi Liu, Fuzhou Tang

**Affiliations:** 1 School of Biology and Engineering, Guizhou Medical University, Guiyang, 550025 People’s Republic of China; 2 Guiyang Maternal and Child Health Care Hospital, Guiyang Children’s Hospital, Guiyang, 550025 People’s Republic of China; 3 Changshun County Medical Group Central Hospital, Guizhou, 550025 People’s Republic of China

**Keywords:** Cancer models, Oncogenes

## Abstract

Alternative splicing (AS) events associated with oncogenic processes present anomalous perturbations in many cancers, including ovarian carcinoma. There are no reliable features to predict survival outcomes for ovarian cancer patients. In this study, comprehensive profiling of AS events was conducted by integrating AS data and clinical information of ovarian serous cystadenocarcinoma (OV). Survival-related AS events were identified by Univariate Cox regression analysis. Then, least absolute shrinkage and selection operator (LASSO) and multivariate Cox regression analysis were used to construct the prognostic signatures within each AS type. Furthermore, we established a splicing-related network to reveal the potential regulatory mechanisms between splicing factors and candidate AS events. A total of 730 AS events were identified as survival-associated splicing events, and the final prognostic signature based on all seven types of AS events could serve as an independent prognostic indicator and had powerful efficiency in distinguishing patient outcomes. In addition, survival-related AS events might be involved in tumor-related pathways including base excision repair and pyrimidine metabolism pathways, and some splicing factors might be correlated with prognosis-related AS events, including SPEN, SF3B5, RNPC3, LUC7L3, SRSF11 and PRPF38B. Our study constructs an independent prognostic signature for predicting ovarian cancer patients’ survival outcome and contributes to elucidating the underlying mechanism of AS in tumor development.

## Introduction

Alternative splicing (AS) is a pre-mRNA processing pathway in which introns are selectively removed to produce functionally distinct mRNAs^1^. It is an important posttranscriptional regulatory mechanism and also serves as the main driving force contributing to proteomic and transcriptome diversity in multicellular eukaryotes^2^. Studies using high-throughput sequencing have confirmed that up to 90% of human genes undergo AS process and generate at least two mRNA isoforms^3^. Mammalian cells can generate transcript variants to better adapt to the environment through AS. However, accumulated evidence revealed aberrant AS events are closely related to various diseases, including spinal muscular atrophy, cystic fibrosis, retinitis pigmentosa, frasier syndrome, growth hormone deficiency and cancer^4,5^. AS can directly participate in the process of regulating tumor proliferation, apoptosis, hypoxia, angiogenesis, immune escape and metastasis^6,7^. Generally, cancer-specific splicing isoforms are orchestrated by limited splicing factors to activate the cancer signaling pathways. The mutation and abnormal expression of splicing factors could lead to global changes in AS behavior^8,9^. Cancer researchers have realized that AS events and splicing factors have the potential to be developed as diagnostic and prognostic biomarkers, as well as the therapeutic targets^10^.

Ovarian carcinoma is one of the most common malignant tumors in the female population, and it is also the deadliest malignancy among the female reproductive tract cancers^11^. The 5-year survival rate for ovarian cancer patients is less than 30% due to the high heterogeneity and lack of effective means for early diagnosis^12^. In ovarian cancer, cancer-specific AS events have been investigated by comparing tumor tissues with normal tissues, and a simple prognosis analysis was conducted to assess AS events^13^. However, there is still a lack of the clinical transfer for the prognostic value of AS, and the role of survival-associated AS events in cancer biology requires further study. Therefore, a systematical investigation of survival-associated AS events in ovarian cancer patients should be performed to build an independent prognostic signature, which could be valuable information for exploiting personalized treatment strategy and therapeutic targets.

In this study, we conducted an in-depth analysis of AS profiling based on ovarian serous cystadenocarcinoma cohort from the Cancer Genome Atlas database, evaluating the survival-associated AS events. Then, the least absolute shrinkage and selection operator (LASSO) Cox regression was used to develop AS-based signatures in seven AS types. More importantly, the final prognostic signature was demonstrated to be an independent prognostic indicator after multivariate adjustment by clinical parameters. In addition, functional enrichment analysis and splicing factor regulatory network were performed. These results may contribute to understand the underlying mechanisms of AS in ovarian cancer progression.

## Results

### Overview of AS events

**Figure 1:**
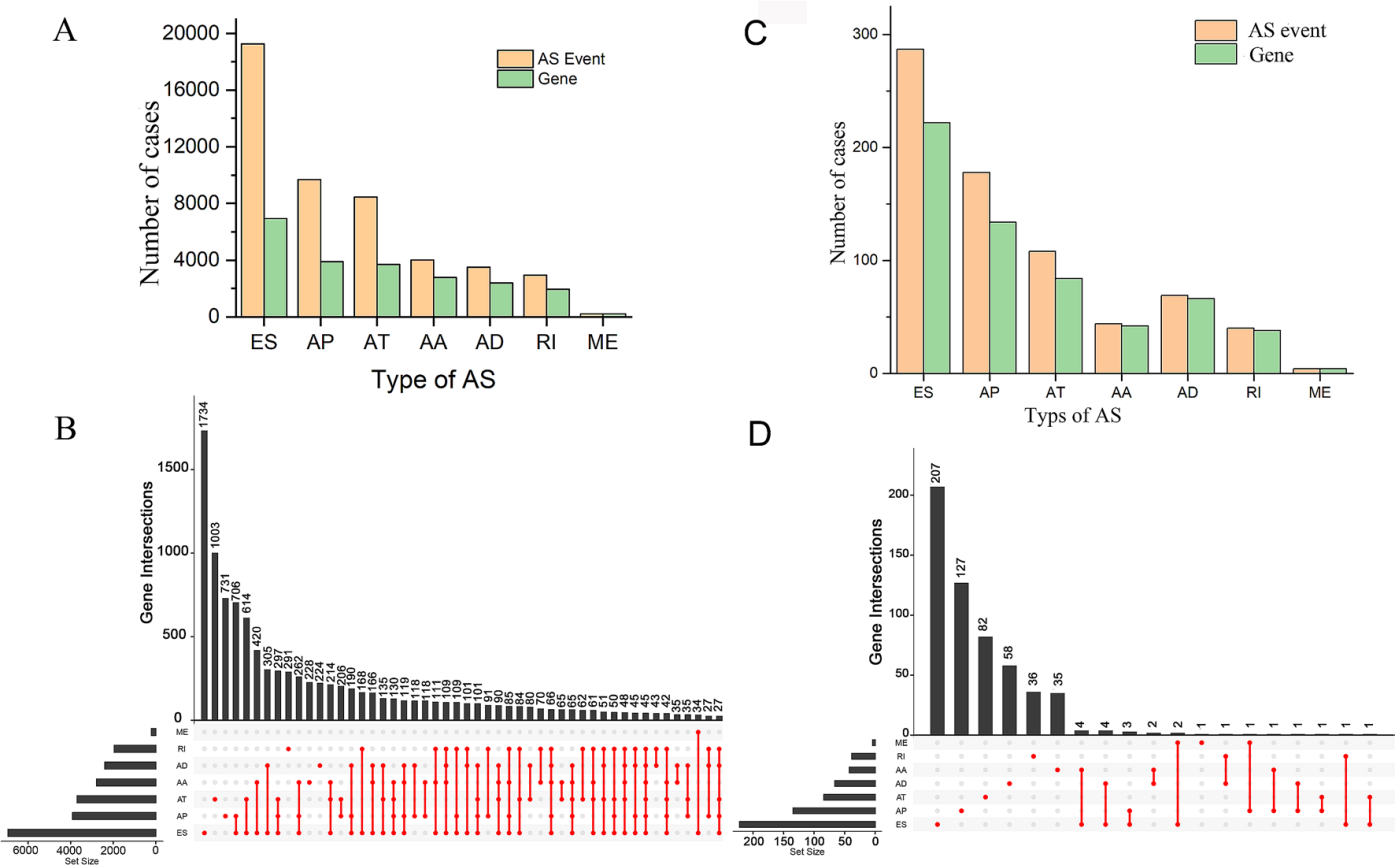
Overview and prognosis-associated AS events in this study. (**A**) Number of AS events and corresponding genes from 397 ovarian carcinoma patients. (**B**) UpSet plots in ovarian carcinoma showing the interactions among the seven types of AS events. One gene may have up to five types of AS events. (**C**) Number of prognosis-associated AS events and corresponding prognosis-associated genes obtained by using univariate COX analysis. (**D**) UpSet plots in ovarian carcinoma showing the interactions among the prognosis-related seven types of AS events. One gene may have up to two types of AS to be related with patient survival.

We processed splice-seq files and clinical information of 397 patients in the present analysis. Seven types of AS events were identified, including Mutually Exclusive Exons (ME), Retained Intron (RI), Alternate Donor site (AD), Alternate Acceptor site (AA), Alternate Terminator (AT), Alternate Promoter (AP), and Exon Skip (ES). As a whole, 48,049 mRNA AS events were detected in 10,581 genes. In detail, we detected 19,251 ES events in 6931 genes, 9689 AP events in 3901 genes, 8453 AT events in 3691 genes, 4006 AA events in 2777 genes, 3497 AD events in 2389 genes, 2946 RI events in 2951 genes and 207 ME events in 201 genes (Fig. 1A,B). It was noteworthy that several types of AS events might present in a single gene, and ES events, as the main type, accounted for almost half of all of the AS events.

### Survival associated AS events

To explore the relationship between AS events and OS of patients with ovarian cancer, univariate Cox regression analysis was performed to assess the prognostic value of AS events. A total of 730 survival-associated AS events (*P* < 0.05) were identified in 568 genes, with the following distribution: 287 ESs in 222 genes, 178 APs in 134 genes, 108 ATs in 84 genes, 44 AAs in 42 genes, 69 ADs in 66 genes, 40 RIs in 38 genes and 4 MEs in 4 genes (Fig. 1C). An UpSet plot was used to generate the visualized intersecting sets shown in Fig. 1D, which illustrated that one protein-coding gene may have two types of survival-associated events. ES events were also the most common survival-associated events, followed by AP and AT events.

### Molecular characteristics of survival-associated AS

**Figure 2:**
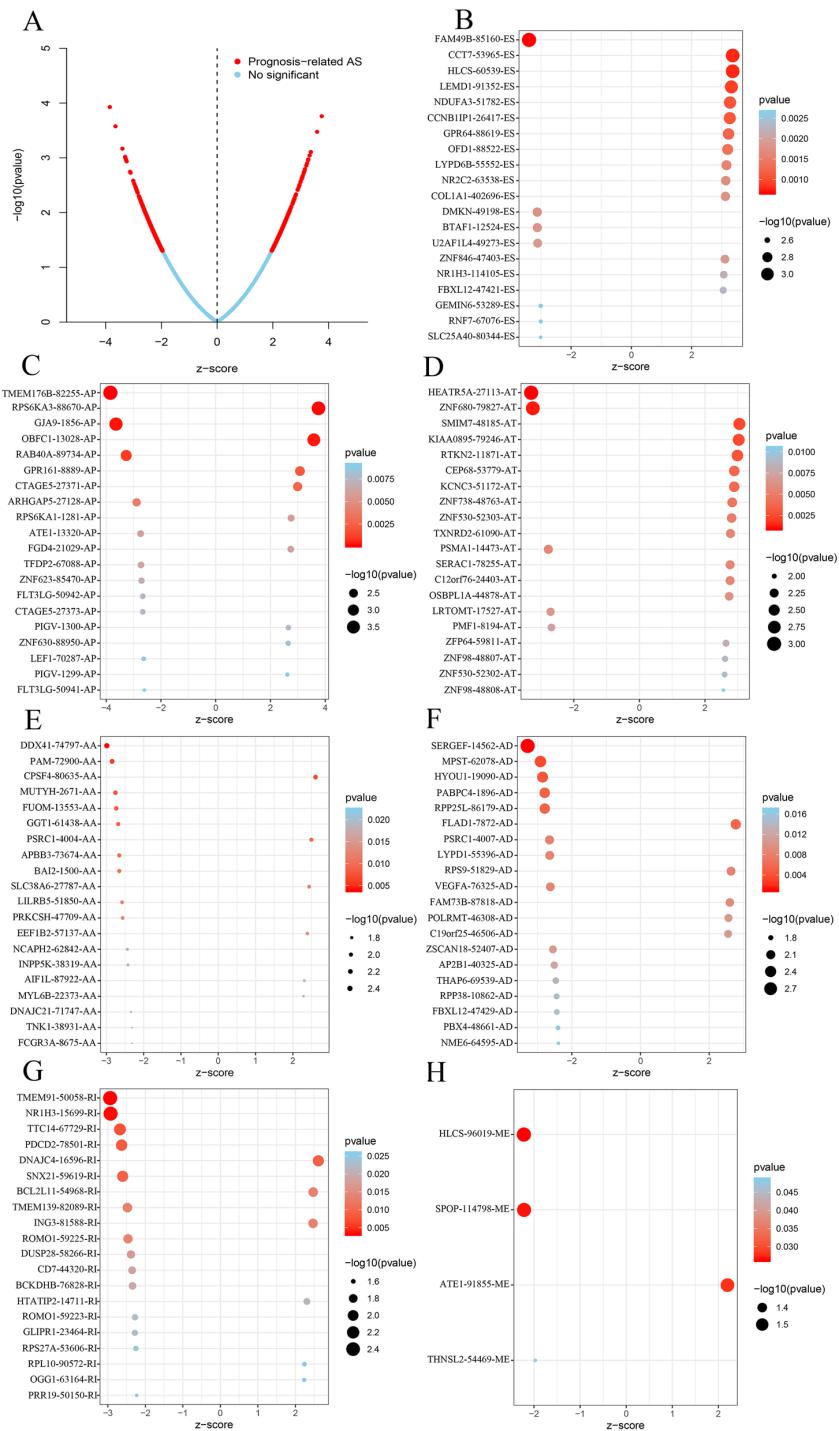
The significant AS events (top 20) in ovarian carcinoma. (**A**) The whole AS events in ovarian carcinoma patients. The red/blue dots showing AS events significantly/without related with prognosis. The top 20 AS events related with clinical outcome based on acceptor sites. The significant survival-related AS events in seven types, including ES (**B**), AP (**C**), AT (**D**), AA (**E**), AD (**F**), RI (**G**), and ME (**H**).

**Figure 3:**
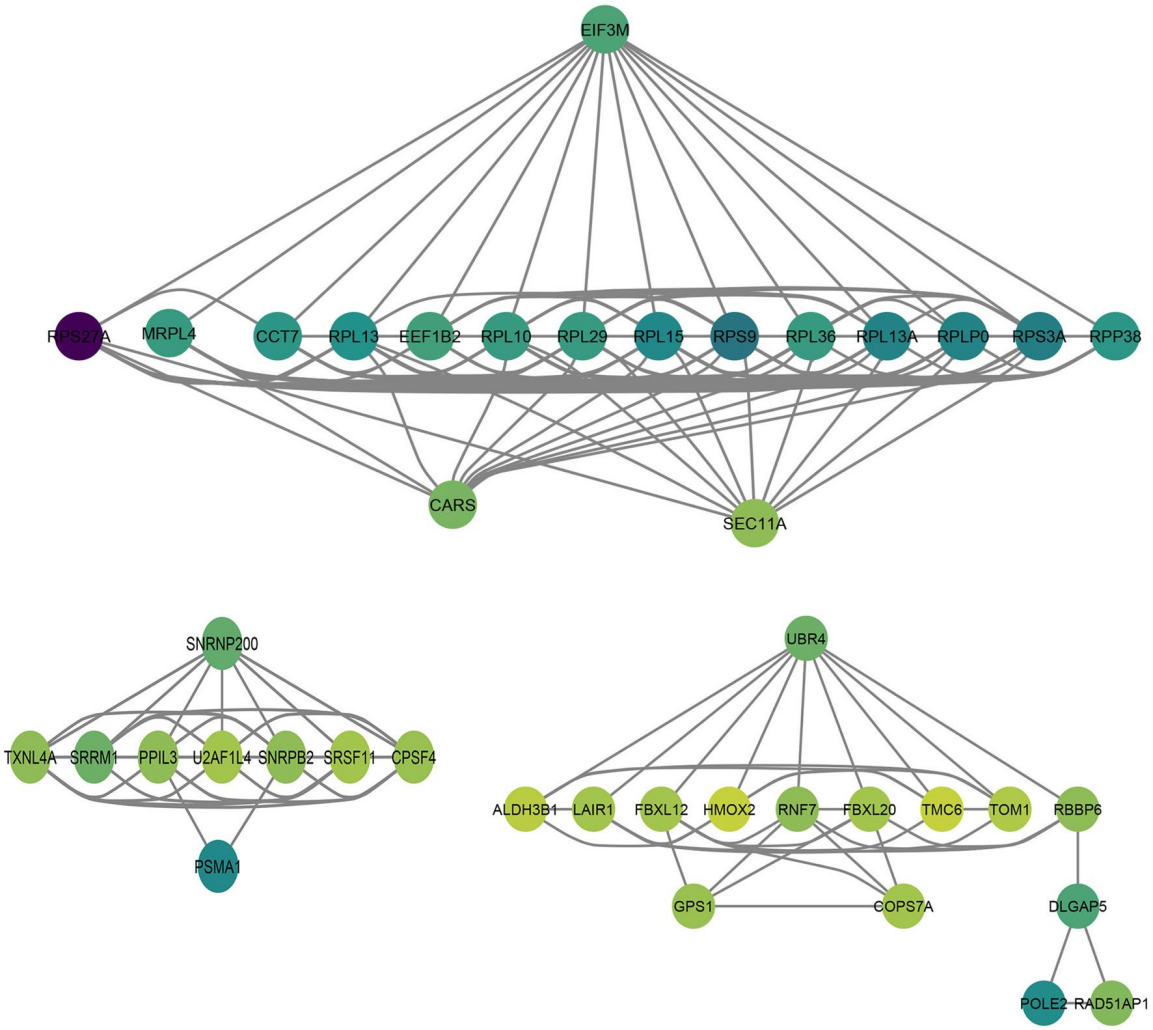
Interaction network of genes with survival-associated AS events in ovarian carcinoma generated by Cytoscape. The more interactive point in the network, the more important it is. Hub genes at EIF3M, RPS27A, SNRNP200 and UBR4 in gene networks.

The distributions of AS events significantly related with patient survival are shown in Fig. 2A. The red dots represent prognosis-associated AS events. The significant survival-related AS events among seven types are also displayed in Fig. 2B–H. Furthermore, several bioinformatics analyses were used to explore the molecular characteristics of gene with survival-related AS events. Reactome was used to reveal the gene interaction networks, and EIF3M, RPS27A, SNRNP200 and UBR4 were found to the hub genes (Fig. 3). The functional annotations of clusterProfiler showed that “translational elongation”, “nitrogen compound catabolic process” and “translation” were the three most effective biological process terms (Table 1A). “Ribonucleoprotein complex”, “ribosome” and “ribosomal subunit” were the three most effective cellular component terms (Table 1B). “Structural constituent of ribosome”, “metal ion binding” and “RNA binding” were the three most significant molecular function terms (Table 1C). The pathway analysis using the Kyoto Encyclopedia of Genes and Genomes (KEGG) pathway database identified the main enriched genes associated with “ribosome”, “base excision repair” and “pyrimidine metabolism” (Table 2).

**Table 1:** Gene ontology analysis of genes with survival-related AS events. Biological process (Table 1A); Cellular component (Table 1B); Molecular function (Table 1C).

A			
ID	Cellular component	*P* value	Cont
GO:0030529	Ribonucleoprotein complex	3.54E−04	30
GO:0005840	Ribosome	3.42E−03	15
GO:0033279	Ribosomal subunit	3.51E−03	11
GO:0005829	Cytosol	4.45E−03	55
GO:0031974	Membrane-enclosed lumen	6.98E−03	71
GO:0022626	Cytosolic ribosome	8.08E−03	8
GO:0031981	Nuclear lumen	1.02E−02	57
GO:0005654	Nucleoplasm	1.08E−02	38

B			
ID	Biological process	*P* value	Count
GO:0006414	Translational elongation	7.26E−04	11
GO:0044270	Nitrogen compound catabolic process	3.31E−03	8
GO:0006412	Translation	3.92E−03	20
GO:0042476	Odontogenesis	4.72E−03	7
GO:0009264	Deoxyribonucleotide catabolic process	7.08E−03	4
GO:0006259	DNA metabolic process	7.34E−03	26
GO:0007017	Microtubule-based process	7.39E−03	16

C			
ID	Molecular functions	*P* value	Cont
GO:0003735	Structural constituent of ribosome	1.30E−03	14
GO:0046872	Metal ion binding	1.12E−02	143
GO:0003723	RNA binding	1.14E−02	33
GO:0008270	Zinc ion binding	1.17E−02	86
GO:0043169	Cation binding	1.18E−02	144
GO:0008017	Microtubule binding	1.84E−02	7
GO:0043167	Ion binding	1.97E−02	144
GO:0003712	Transcription cofactor activity	2.02E−02	19

**Table 2:** The analysis of Kyoto Encyclopedia of Genes and Genomes pathway with genes in survival-associated AS events (the pathway analysis using the Kyoto Encyclopedia of Genes and Genomes (KEGG) pathway database^31–33^).

Term	Gene rate (%)	P value	Cont
Ribosome	1.86	6.12E−04	10
Base excision repair	0.93	1.52E−02	5
Pyrimidine metabolism	1.30	4.74E−02	7
Non-homologous end-joining	0.56	4.87E−02	3
Glyoxylate and dicarboxylate metabolism	0.56	6.33E−02	3
Riboflavin metabolism	0.56	7.11E−02	3

### Prognostic signatures for ovarian carcinoma patients

**Figure 4:**
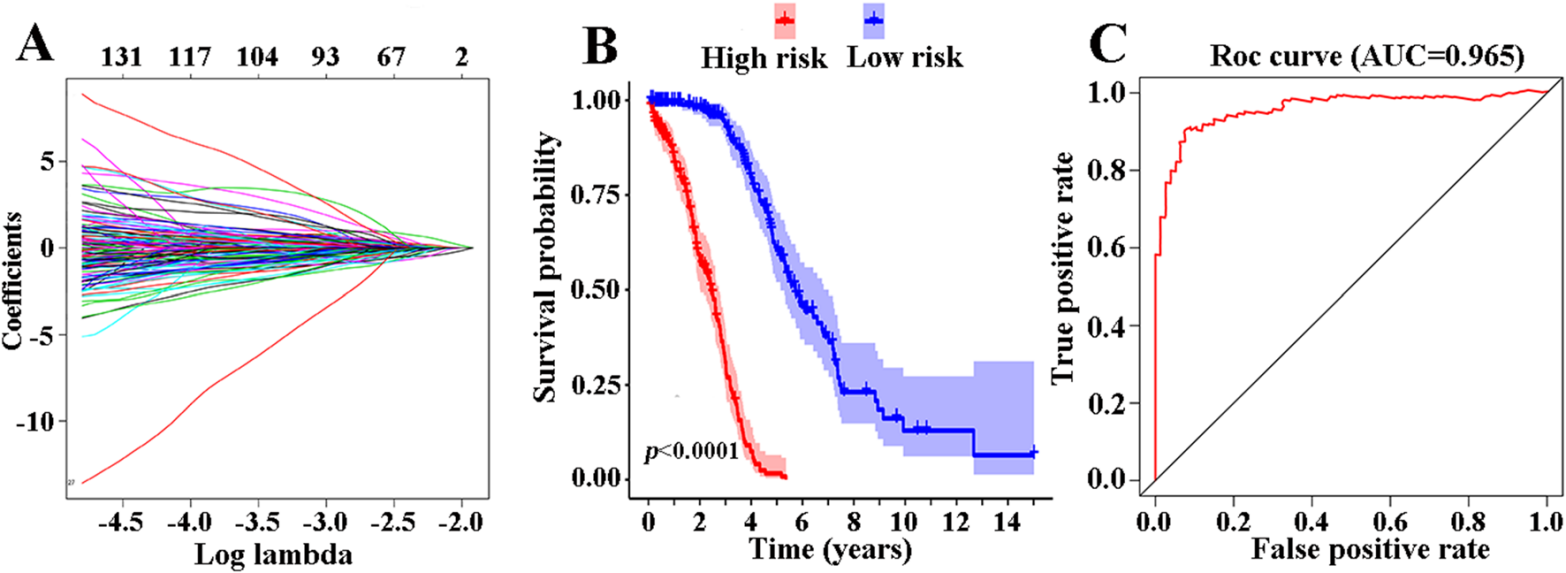
Prognostic signatures for ovarian carcinoma patients. (**A**) LASSO COX analysis in seven types of AS events. (**B**) Kaplan–Meier curves of prognostic predictors for ovarian carcinoma in seven types of AS events. (**C**) ROC curves of prognostic predictors for ovarian carcinoma in seven types of AS events.

**Figure 5:**
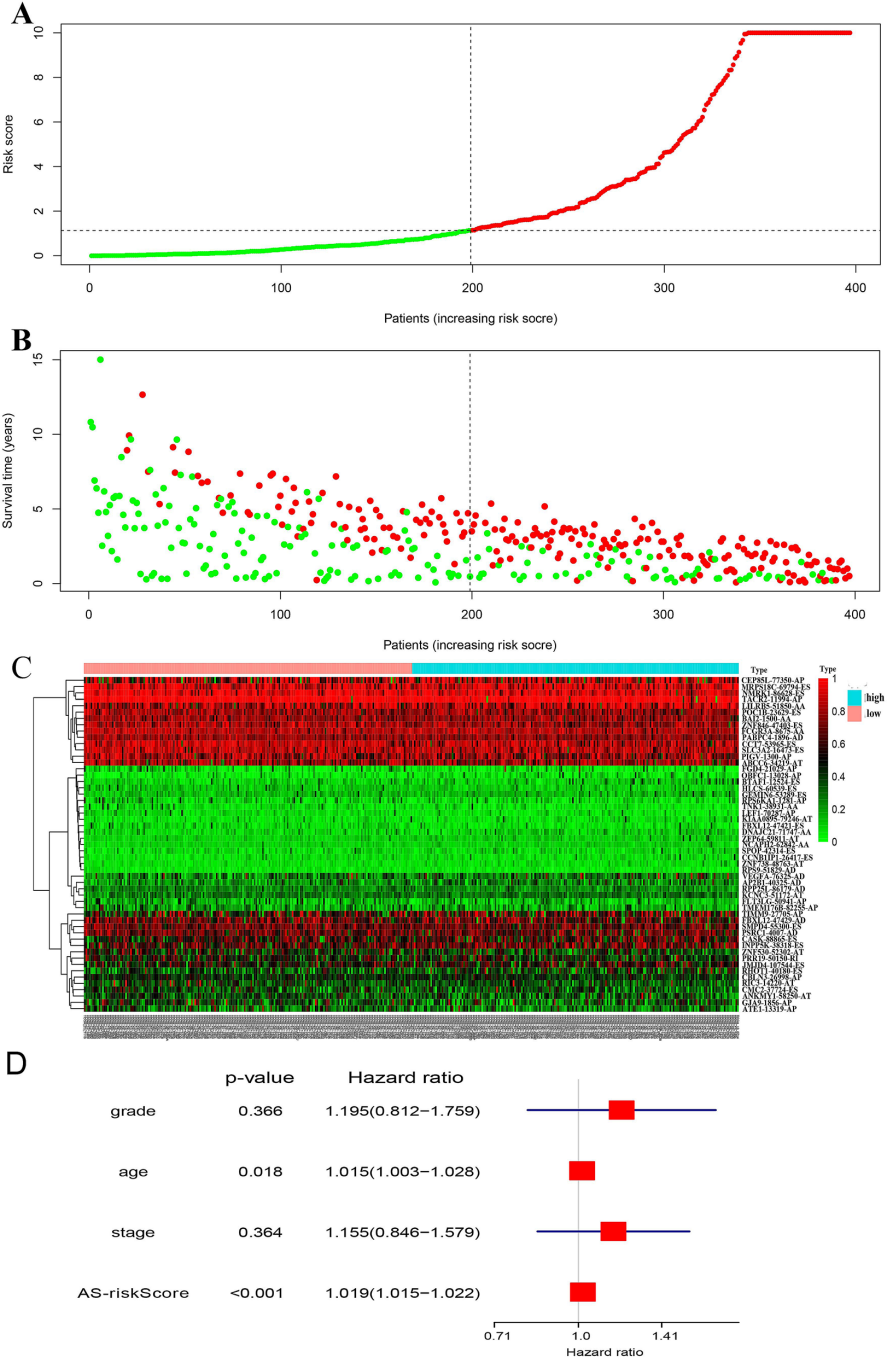
The recognition capability of prognostic signature for dividing patients into low- and high-risk groups in ovarian carcinoma. (**A**) The risk values based on AS in low-risk (green dots) and high-risk (red dots) patients. (**B**) Survival time for ovarian carcinoma patients. The left and right respectively showing low-risk and high-risk patients. Green dots indicating survival patients and red dots indicating dead patients. (**C**) The PSI values of final prognostic signature in ovarian patents. (**D**) The independent prognostic indicator for AS events.

To screen out prognostic predictors for patient survival, the significant survival-associated AS events in the seven types were selected as candidates. The least absolute shrinkage and selection operator (LASSO) Cox analysis were used to build the prognostic signature, based on ME, RI, AD, AA, AT, AP and ES (Fig. 4A and Supplementary Fig. 1). Interestingly, all seven prognostic models showed great prognostic value (Fig. 4B and Supplementary Fig. 2). Furthermore, ROC curves examined the predictive accuracy of the models (Fig. 4C and Supplementary Fig. 3). The AUC value of the final prognostic signature that integrated all types was 0.965 followed by the AA model with an AUC value of 0.862, the ES model with an AUC value of 0.855, and the AP model with an AUC of 0.811. The final prognostic signature is an ideal predictor (Fig. 5A), and could significantly distinguish the ovarian cancer patients with different clinical outcomes (Fig. 5B). Figure 5C shows the PSI values of AS events for building the final prognostic signature. After multivariate adjustment by clinical parameters, the final prognostic signature can still act as an independent prognostic indicator (HR = 1.019, 95% CI: 1.015–1.022, *P* < 0.001; Fig. 5D).

### Survival-associated splicing regulatory network

**Figure 6:**
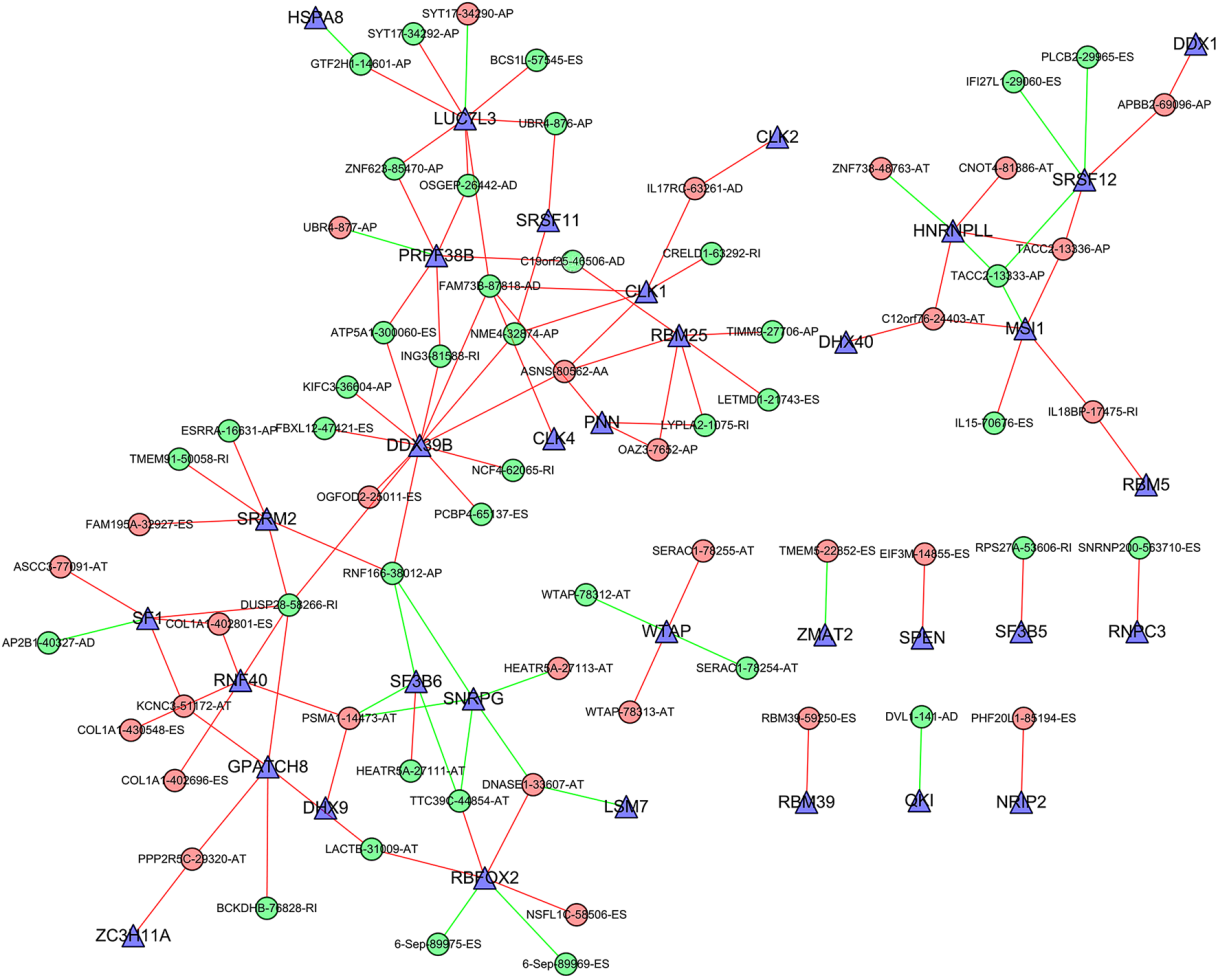
Splicing correlation network in ovarian carcinoma. AS events were negatively/positively associated with survival times representing with red/green dots, and blue triangles were survival-related splicing factors.

AS is orchestrated by splicing factors, which recognize and bind to pre-mRNAs at specific positions. To explore whether the prognosis-related AS events are modulated by specific splicing factors in ovarian cancer, we constructed a splicing-regulatory network (Fig. 6). The expression of 32 splicing factors (triangle nodes) were significantly correlated with survival-associated AS events, and most of them were positively correlated. Notably, A single splicing factor might regulate multiple survival-related AS events. For example, splicing factor LUC7L3 could regulate the AP events of SYT17 (34,290), UBR4 (876), ZNF623 (85,470), GTF2H1 (14,601) and SYT17 (34,292) and ES events of BCS1L (57,545) and AD events of OSGEP (26,442). Splicing factor PRPF38B might regulate the AP event of UBR4 (877) and ZNF623 (85,470) and AD events of C19orf25 (46,506) and OSGEP (26,442) and RI event of ING3 (81,588) and ES event of ATP5A1 (300,060).

### Discussion

AS of pre-mRNA as a posttranscriptional process for gene modification generates many mRNA and protein isoforms with diverse regulatory and functional properties^1,2^. In addition, the splicing isoforms of specific genes act as the drivers of cancer, which are related to tumor development, proliferation, metastasis, survival and drug resistance^6,7^. However, there are still many unanswered questions about the role of AS events in ovarian carcinoma due to the complexity and diversity of molecular functions, as well as the lack of available large-sample public AS profiles and systematic analyses of AS events. In this paper, several biomedical computational approaches were adopted to analyze the AS events by integrated use of AS event profiles and the clinical outcomes of ovarian carcinoma patients. An excellent prognostic model was constructed that was able to divide ovarian carcinoma patients into different subgroups according to their distinct survival outcomes. In particular, we found that AS events could be used as an independent prognostic factor.

Ovarian carcinoma is one of the deadliest tumors among female patients, with a 5-year survival rate of less than 30%^11,12^. Previous studies have identified multiple markers of potential drug targets that may help improve the survival rate of ovarian carcinoma patients such as WFDC2, CA125 and MSLN^14–16^. In recent years, with the development of sequencing technology, vast quantities of information from whole-genome or transcriptome analyses has been gathered into the TCGA data. Interestingly, the exploration of genome AS in SpliceSeq analyses presented a distinct correlation between AS events and the prognosis of some types of cancer, including glioblastoma, gastrointestinal cancer, bladder cancer, non-small cell lung cancer, breast cancer and ovarian carcinoma^13,17–19^. It should be noted that although Zhu et al. have reported the survival-related AS events in ovarian carcinoma^13^, systematic survival analyses of AS events in ovarian cancer is still needed. Our study could be considered more extensive for the following reasons. Firstly, we only selected the appropriate patients (We excluded the patients with an overall survival of less than 30 days, and the cases with more than 20% missing AS events were excluded) and AS events to accurately identify survival-related AS events (The AS events with PSI value > 75% were chosen, and then AS events were excluded with standard deviation < 0.01). Secondly, we identified more accurate and reliable hub genes including EIF3M, RPS27A, SNRNP200 and UBR4 (Fig. 3), and we also performed an enrichment analysis to characterize the role of AS in ovarian cancer. Thirdly, we used multiple algorithms (including univariate Cox, multivariate Cox and Lasso regression) to build a more reliable prognostic model. The final prognostic signature was proved to be an independent predictor and has great clinical application value. Finally, based more accurate survival-related AS events, we identified more accurate and reliable splicing factors including SPEN, SF3B5, RNPC3, LUC7L3, SRSF11 and PRPF38B (Fig. 6).

The LASSO Cox regression mode was used to construct the prognostic signature. Our results showed that the final model by integrating seven types of AS events could significantly distinguish patients with different clinical outcomes (the 5-year survival rates were 61.50% and 0.01% in the low risk and high risk groups, respectively) and had the highest reliable efficiency (the AUC value of the ROC was 0.965). Thus, AS events could be used as an ideal prognostic signature for predicting the clinical outcomes of ovarian carcinoma patients. Furthermore, we found that the final prognostic signature could act as stable and independent predictor after multivariate adjustment by clinical parameters (Fig. 5D), which provides a more accurate and convenient way to predict the survival of ovarian cancer patients.

Several genes associated with aberrant AS in ovarian carcinoma have been found in previous studies. For example, a special splice variant of EVI1 plays a potential role in modulating the initiation and progression of ovarian carcinoma^20^. CD44v8–10, a CD44 variant including exons v8-10, is related to the prognosis and metastasis of ovarian carcinoma^21^. Multiple splicing isoforms of HE4 exhibit differences in regulation and expression in both normal and ovarian carcinoma tissues^22^. In addition, the microarray study has detected aberrant AS of genes in ovarian carcinomas, including FGFR2, DNNP3B, KITLG, MDM2 and MRP1^23^. In this paper, we identified the potential gene with prognosis-related AS event in ovarian carcinomas (the multiple genes presented in the network), and EIF3M, RPS27A, SNRNP200 and UBR4 were found at the core of gene interaction network. Furthermore, functional analysis revealed that these genes were actively participant in three important signaling pathways (“Ribosome”, “base excision repair” and “pyrimidine metabolism”) to influence the clinical outcomes of ovarian cancer patients. Interestingly, EIF3M is relevant to endometrial carcinogenesis, and the gene also has a modulation role among tumorigenesis-related genes in colon cancer^24^; RPS27A which has been identified as an important prognostic gene in hepatocellular carcinoma, has also been used as a critical biomarker for predicting the metastasis and development of gastric cancer^25^; UBR4 mediates the ubiquitylation of methionine adenosyltransferase IIa, which regulates the growth of hepatocellular cancer, and is is also involved in the prognosis of triple-negative breast cancer^26^.

AS events are mainly orchestrated by a limited number of splicing factors, which bind to pre-mRNAs regulating the selection of splicing site^9^. Growing body of evidence has shown that the global change of splicing behavior in cancer is driven by abnormal expression or mutation of splicing factors. For example, the splicing factor SF2/ASF regulates AS of S6K1, inducing oncogenic properties in most human tumors^27^, and the SF3B2 modulated androgen receptor splice variant-7 is related to human prostate cancer progression^28^. Our splicing regulation network analysis showed some splicing factors might be correlated with prognosis-related AS events, including SPEN, SF3B5, RNPC3, LUC7L3, SRSF11 and PRPF38B (Fig. 6), suggesting that these splicing factors could play crucial roles in ovarian cancer development. In previous studies, researchers have found that the overexpression of SPEN was involved in drug responsiveness in breast cancer^29^, and significant downregulation of SF3B5 was revealed in acute myeloid leukemia patients^30^. However, the roles of these splicing factors in ovarian carcinomas still need to be further tested. Our results also showed that several AS events could be modulated by a single splicing factor, and diverse splicing factors might regulate the same AS events, implying the variation of AS behavior requires cooperative action of splicing factors.

This paper has some limitations due to the following aspects. Firstly, we did not find other independent cohorts with a large number of OV samples in the public resource for independent cohort validation. Secondly, experiments (in vivo and in vitro) are needed to elucidate the biological functions of AS events and splicing factors. Especially, experimental validation these potential splicing factors (e.g. SPEN, SF3B5, RNPC3, LUC7L3, SRSF11, and PRPF38B) regulate alternative splicing events, and it is also important to use experimental validation of wet analysis (RT-PCR) and protein level, and consider the fact that different portions of the tumour have different expression and splicing profiles as well as the importance of the micro-environment.

## Conclusions

In summary, we build an excellent prognostic model for predicting clinical outcomes, and demonstrated that it could be used as an independent predictor for ovarian carcinoma. Furthermore, we constructed a gene interaction network with survival-associated AS events and a correlation network between splicing factors and AS events. These results will help develop personalized treatment options and new therapeutic targets for patients with ovarian cancer.

## Materials and methods

### Assortment of AS event data

RNA-seq data of ovarian serous cystadenocarcinoma cohorts was obtained from the TCGA and the SpliceSeq tool was used to analyze the transcript splicing patterns. Information on Percent Spliced In (PSI) was used to quantify AS events and was selected for calculation of the seven types of AS events. The PSI value showed a shift in splicing events ranging from zero to one. The AS events with PSI value > 75% were chosen. Clinical data of ovarian carcinoma patients were also obtained and abstracted from the pan-cancer atlas database of TCGA.

### Survival analysis and prognostic signature construction

A total of 397 ovarian carcinoma patients were selected for the survival analysis. We excluded the patients with an overall survival of less than 30 days, and the cases with more than 20% missing AS events were excluded. Besides, in order to accurately identify prognostic-related AS events, we also excluded the AS events with standard deviation < 0.01. Univariate Cox regression analysis was used to evaluate the association between the PSI value of each AS event and the overall survival of ovarian carcinoma patients. In seven types of AS events, survival-related AS events were analyzed by LASSO regression Cox analysis to produce prognostic signatures. Additionally, multiplying the PSI values and the coefficient from LASSO Cox analysis were used to analyze the prognostic signatures for overall survival prediction. The time dependent receiver-operator characteristic (ROC) curve was constructed using the survival ROC package in R software. The area under the curve (AUC) of the ROC curve was used to evaluate the effectiveness of the prognostic signatures. The subsequent Kaplan–Meier curves were also plotted for distinguishing the low- and high-risk of ovarian carcinoma patients, and the Log-rank test was used to analyze the differences between the two groups. All reported *P* values were two-sided, and all analyses were carried out using R/Bioconductor. Finally, To assess whether AS events could be used as an independent Predictor, the following clinical parameters were also evaluated by multivariable Cox regression analysis, including age (≥ 50 and < 50), AJCC TNM stage (stage III/IV or stage I/II), grade (III/I–II), and AS risk score (High-risk/Low-risk).

### Gene network construction and functional annotation

Cytoscape's Reactome was used to construct the gene network, exploring the important hub genes of survival-related AS events. Furthermore, the functional categories of the hub genes were also analyzed by the KEGG^31–33^ (KEGG permission document shown in supplementary file) and Gene ontology (GO) based on the standards of a *P*-value of < 0.05.

### Splicing correlation network construction

The expression of splicing factor genes was curated from level-3 mRNA data in the TCGA dataset. Then, the regulatory network of splicing factors associated with AS events was constructed by Cytoscape (version 3.7.1) according to the correlation between the PSI values of the prognosis-associated AS events and the expression values of the splicing factors genes. *P* value of < 0.001 and correlation coefficient value of > 0.4 were considered significant.

### Ethical approval

All data in this paper was obtained from public database (the cancer genome atlas (TCGA), TCGA SpliceSeq, and the pan-cancer atlas database of TCGA).

## Supplementary information

Supplementary Figures.
