# Supplementary material for: Alternative splicing acts as an independent prognosticator in ovarian carcinoma
Source: Sci Rep. 2021 May 17;11:10413. doi: 10.1038/s41598-021-89778-0 (PMC8129203; doi:10.1038/s41598-021-89778-0)
Supplement: Supplementary file 1 — Supplementary Figures. [file 41598_2021_89778_MOESM1_ESM.pdf]

Title: Alternative splicing acts as an independent prognosticator in ovarian carcinoma

Yi Liu [1], Kaide Xia [1, 2], Xue Yang [1], Shichao Zhang [2], Li Wang [3], Shan Ren [1], Houming Zhou [2], Fuzhou Tang [2]* , Yan Ouyang [2*]

Running title: Alternative splicing as an independent prognosticator

[1]Guiyang Maternal and Child Health Care Hospital, Guiyang Children's Hospital, Guiyang 550025, P. R. China.
[2]School of Biology & Engineering, Guizhou Medical University, Guiyang 550025, P. R. China.
[3]Changshun County Medical Group Central Hospital, Guizhou, 550025, P. R. China.

*Corresponding Author: Fuzhou Tang or Yan Ouyang.
Email: tangfuzhou@163.com or 287409290@qq.com

Yi Liu and Kaide Xia contributed equally to this work.

Supplementary Figure 1. LASSO COX analysis based on AS events. LASSO COX analysis in seven types of AS events (A), ES (B), AP (C), AT (D), AA (E), AD (F), RI (G), and ME (H).

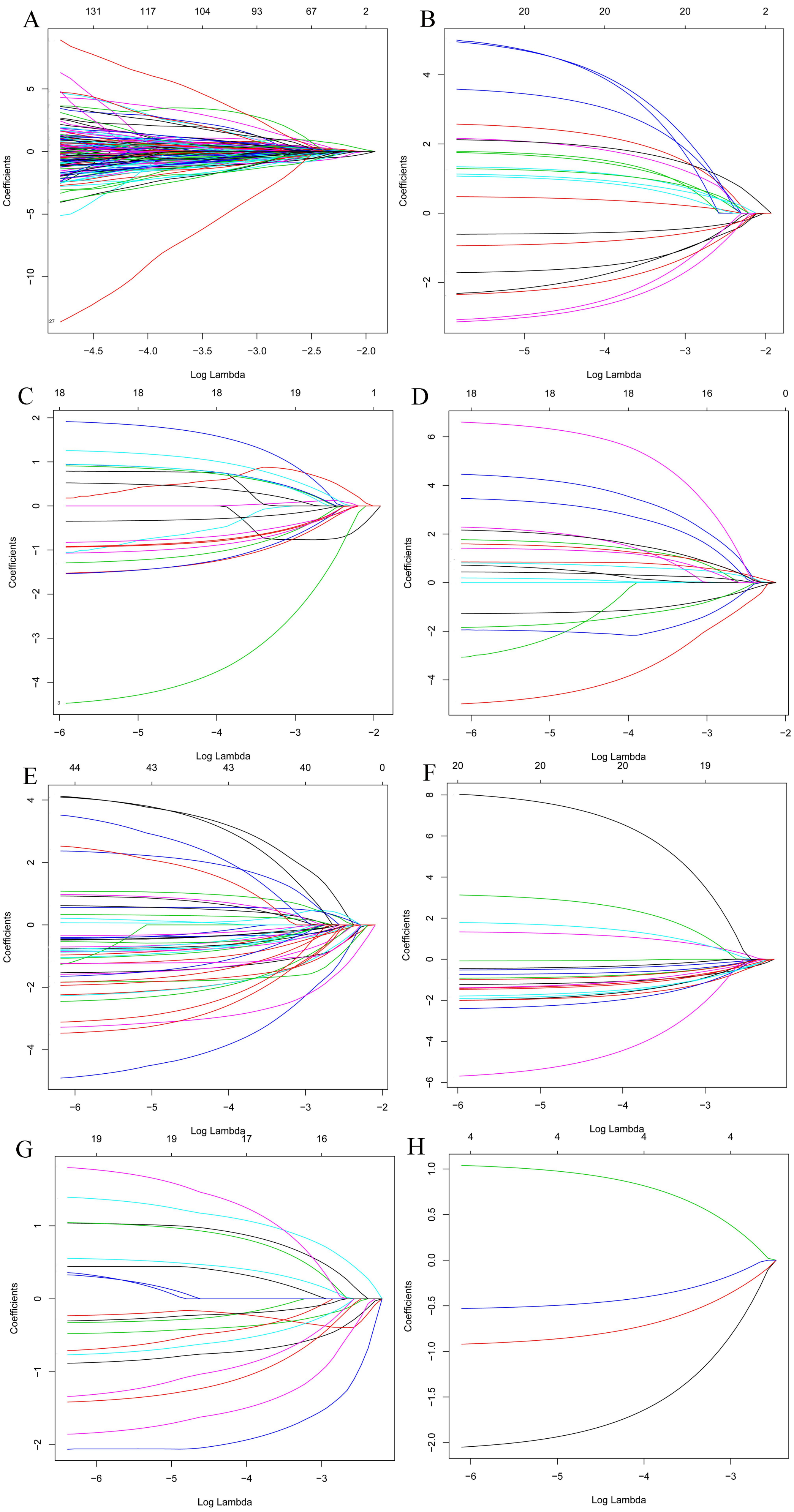

A
131
117
104
93
67
2
Coefficients
5
0
−5
−10
−4.5
−4.0
−3.5
−3.0
−2.5
−2.0
Log Lambda
B
20
20
20
2
4
2
0
−2
−5
−4
−3
−2
Coefficients
Log Lambda
C
18
18
18
19
1
2
1
0
−1
−2
−3
−4
−6
−5
−4
−3
−2
Coefficients
Log Lambda
D
18
18
18
16
0
6
4
2
0
−2
−4
−6
−5
−4
−3
−2
Coefficients
Log Lambda
E
44
43
43
40
0
4
2
0
−2
−4
−6
−5
−4
−3
−2
Coefficients
Log Lambda
F
20
20
20
19
8
6
4
2
0
−2
−4
−6
−6
−5
−4
−3
Coefficients
Log Lambda
G
19
19
17
16
1
0
−1
−2
−6
−5
−4
−3
Coefficients
Log Lambda
H
4
4
4
4
1.0
0.5
0.0
−0.5
−1.0
−1.5
−2.0
−6
−5
−4
−3
Coefficients
Log Lambda

Supplementary Figure 2. Kaplan-Meier curves of prognostic predictors for ovarian carcinoma. Kaplan-Meier curves in seven types of AS events (A), ES (B), AP (C), AT (D), AA (E), AD (F), RI (G), and ME (H).

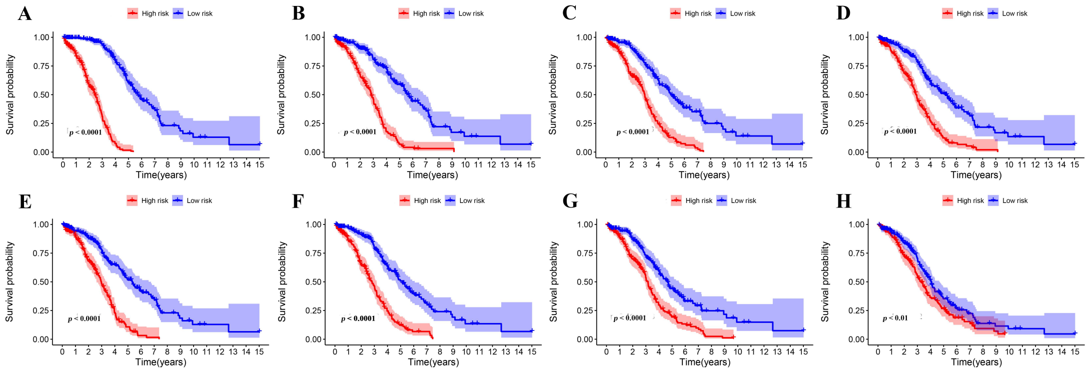

Supplementary Figure 3. ROC curves of prognostic predictors for ovarian carcinoma. ROC curves in seven types of AS events (A), ES (B), AP (C), AT (D), AA (E), AD (F), RI (G), and ME (H).

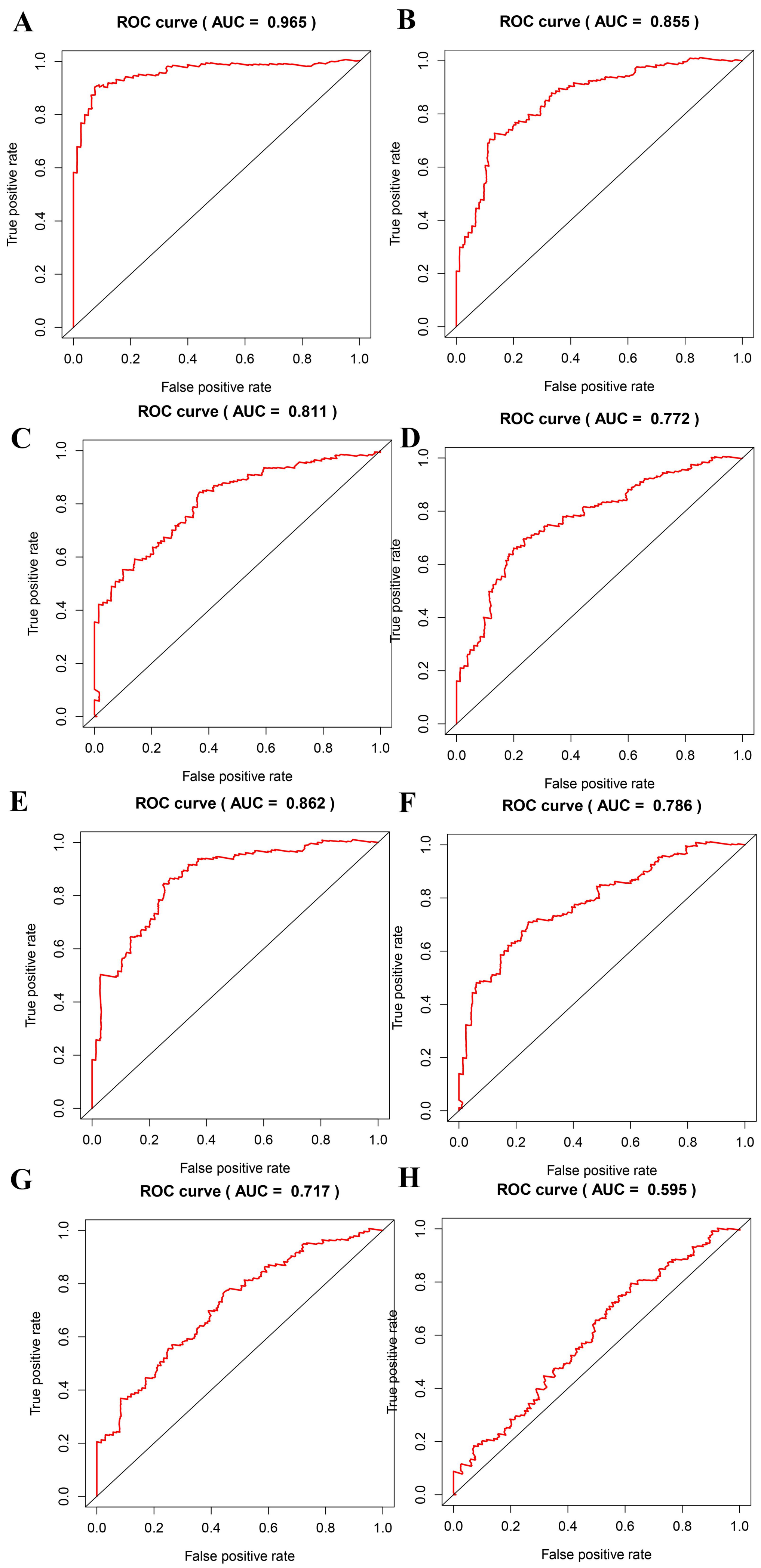
A
ROC curve (AUC = 0.965)
B
ROC curve (AUC = 0.855)
C
ROC curve (AUC = 0.811)
D
ROC curve (AUC = 0.772)
E
ROC curve (AUC = 0.862)
F
ROC curve (AUC = 0.786)
G
ROC curve (AUC = 0.717)
H
ROC curve (AUC = 0.595)
True positive rate
False positive rate
0.0
0.2
0.4
0.6
0.8
1.0
